# Supplementary material for: Innovative Cost-Effective Nano-NiCo2O4 Cathode Catalysts for Oxygen Reduction in Air–Cathode Microbial Electrochemical Systems
Source: Int J Environ Res Public Health. 2022 Sep 15;19(18):11609. doi: 10.3390/ijerph191811609 (PMC9517631; doi:10.3390/ijerph191811609)
Supplement: Supplementary file 1 [file ijerph-19-11609-s001.zip › ijerph-1884573-supplementary.pdf]

Supplementary information

Innovative cost-effective nano-$NiCo_2O_4$ cathode catalysts for oxygen reduction in air-cathode microbial electrochemical systems

Qixing Zhou*, Ruixiang Li, Xiaolin Zhang, Tian Li

MOE Key Laboratory of Pollution Processes and Environmental Criteria / Tianjin Key Laboratory of Environmental Remediation and Pollution Control, College of Environmental Science and Engineering, Nankai University, No. 38 Tongyan Road, Jinnan District, Tianjin 300350, China

* Corresponding author, e-mail: zhouqx@nankai.edu.cn

Table of Contents

Figure S1. The linear sweep voltammetry of the cathodes with 0, 2%, and 5% $NiCo_2O_4$.

Figure S2. Cyclic voltammetry of the cathodes with 0, 2%, 5% $NiCo_2O_4$ under 10 mV/s.

Table S1. The published data of different photocatalysts and their performance.

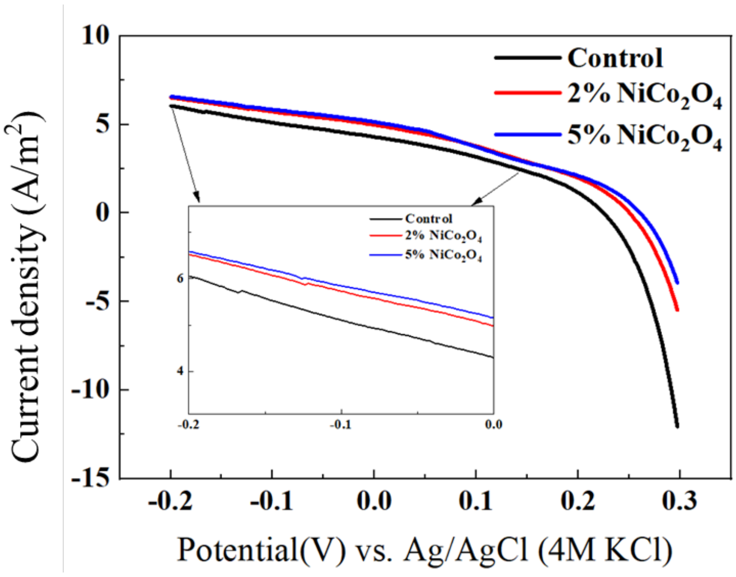

Figure S1. The linear sweep voltammetry of the cathodes with 0, 2%, and 5% $NiCo_2O_4$.

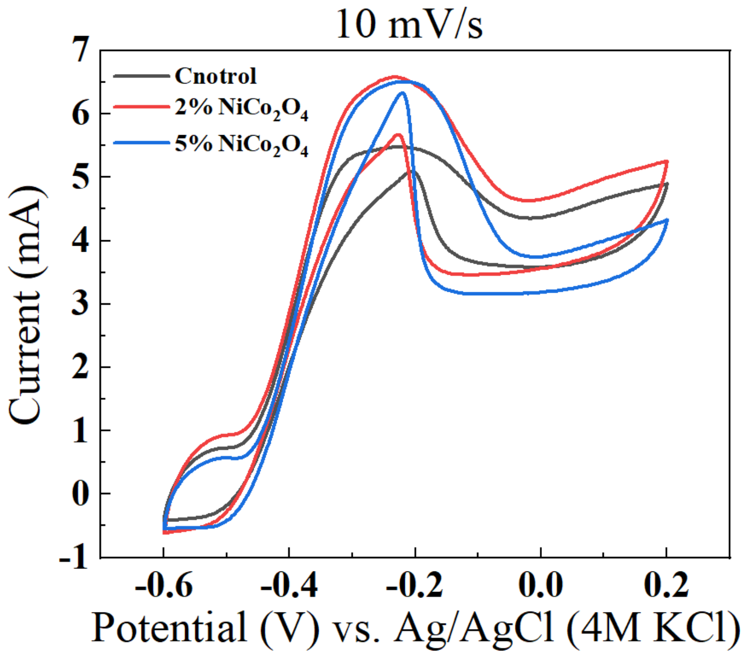

Figure S2. Cyclic voltammetry of the cathodes with 0, 2%, 5% $NiCo_2O_4$ under 10 mV/s.

Table S1. The published data of different photocatalysts and their performance.

MFC	Anode materials	Cathode materials	Cathode catalysts	Power density	Ref.
Dual-chamber MFC	Carbon felt/ stainless steel	Carbon felt/ stainless steel	Rhodium	9.36 W/m^3	[1]
Air-cathode MFC	Carbon cloth	Carbon cloth	Polypyrrole/ carbon black	401.8 mW/m^2	[2]
Single chamber MFC	Carbon cloth	Carbon cloth	Polyaniline/ carbon black/ iron phthalocyanine	630.5 mW/m^2	[3]
Air-cathode MFC	Carbon felt	Carbon felt	SnO_2/ Polyaniline	65 mW/m^2	[4]
Dual-chamber MFC	Carbon felt	Carbon cloth	α-Fe_2O_3/polyaniline	1502.78 mW/m^2	[5]
Air-cathode MFC	Carbon felt	AC/ stainless steel	Co/N-C	2514 mW/m^2	[6]
Single chamber MFC	Carbon fiber brush	Graphitic carbon	AgNPs/Fe_3O_4	1712 mW/m^2	[7]
Single chamber MFC	stainless steel wire mesh	stainless steel wire mesh	V_2O_5/rGO	533 mW/m^2	[8]
Single	Carbon felt	Carbon cloth	GO/MgO	755.63	[9]

chamber MFC				mW/m^2	
Single chamber MFC	Graphite felts	Carbon cloth	cobalt oxide/N/ graphene	713.6 mW/m^2	[10]
Single chamber MFC	Carbon felt	Stainless-steel mesh	$CoFe_2O_4$	1771 mW/m^2	[11,12]

References for supporting information

1. Bhowmick, G.D.; Das, S.; Adhikary, K.; Ghangrekar, M.M.; Mitra, A. Using rhodium as a cathode catalyst for enhancing performance of microbial fuel cell. *Int. J. Hydrog. Energy* **2019**, *44*, 22218-22222, https://doi.org/10.1016/j.ijhydene.2019.06.063.
2. Yuan, Y.; Zhou, S.G.; Zhuang, L. Polypyrrole/carbon black composite as a novel oxygen reduction catalyst for microbial fuel cells. *Journal of Power Sources* **2010**, *195*, 3490-3493, https://doi.org/10.1016/j.jpowsour.2009.12.026.
3. Yuan, Y.; Ahmed, J.; Kim, S. Polyaniline/carbon black composite-supported iron phthalocyanine as an oxygen reduction catalyst for microbial fuel cells. *Journal of Power Sources* **2011**, *196*, 1103-1106, https://doi.org/10.1016/j.jpowsour.2010.08.112.
4. Tiwari, A.K.; Jain, S.; Mungray, A.A.; Mungray, A.K. SnO2:PANI modified cathode for performance enhancement of air-cathode microbial fuel cell. *J. Environ. Chem. Eng.* **2020**, *8*, 8, https://doi.org/10.1016/j.jece.2019.103590.
5. Li, M.; Zhou, S.Q. alpha-Fe2O3/polyaniline nanocomposites as an effective catalyst for improving the electrochemical performance of microbial fuel cell. *Chem. Eng. J.* **2018**, *339*, 539-546, https://doi.org/10.1016/j.cej.2018.02.002.
6. Yang, T.; Li, K.; Pu, L.; Liu, Z.; Ge, B.; Pan, Y.; Liu, Y. Hollow-spherical Co/N-C nanoparticle as an efficient electrocatalyst used in air cathode microbial fuel cell. *Biosens. Bioelectron.* **2016**, *86*, 129-134, https://doi.org/10.1016/j.bios.2016.06.032.
7. Ma, M.; You, S.; Gong, X.; Dai, Y.; Zou, J.; Fu, H. Silver/iron oxide/graphitic carbon composites as bacteriostatic catalysts for enhancing oxygen reduction in microbial fuel cells. *Journal of Power Sources* **2015**, *283*, 74-83, https://doi.org/10.1016/j.jpowsour.2015.02.100.
8. Noori, M.T.; Mukherjee, C.K.; Ghangrekar, M.M. Enhancing performance of microbial fuel cell by using graphene supported V2O5-nanorod catalytic cathode. *Electrochim. Acta* **2017**, *228*, 513-521, https://doi.org/10.1016/j.electacta.2017.01.016.
9. Xin, S.S.; Shen, J.G.; Liu, G.C.; Chen, Q.H.; Xiao, Z.; Zhang, G.D.; Xin, Y.J. Electricity generation and microbial community of single-chamber microbial fuel cells in response to Cu2O nanoparticles/reduced graphene oxide as cathode catalyst. *Chem. Eng. J.* **2020**, *380*, 9, https://doi.org/10.1016/j.cej.2019.122446.
10. Cao, C.; Wei, L.; Su, M.; Wang, G.; Shen, J. Enhanced power generation using nano cobalt oxide anchored nitrogen-decorated reduced graphene oxide as a high-performance air-cathode electrocatalyst in biofuel cells. *RSC Adv.* **2016**, *6*, 52556-52563, https://doi.org/10.1039/c6ra11095a.
11. Zhou, Q.; Ma, S.; Zhan, S. Superior photocatalytic disinfection of Ag-3D ordered mesoporous CeO_2 under visible light condition. *Appl. Catal. B Environ.* 2018, 224, 27–37. https://doi.org/10.1016/j.apcatb.2017.10.032

12. Huang, Q.S.; Zhou, P.J.; Yang, H.; Zhu, L.L.; Wu, H.Y. In situ generation of inverse spinel CoFe2O4 nanoparticles onto nitrogen-doped activated carbon for an effective cathode electrocatalyst of microbial fuel cells. *Chem. Eng. J.* **2017**, *325*, 466-473, https://doi.org/10.1016/j.cej.2017.05.079.
